# Morphometry of Intracranial Carotid Artery Calcifications in Patients with Recent Cerebral Ischemia

**DOI:** 10.3390/jcm14103274

**Published:** 2025-05-08

**Authors:** Bernhard P. Berghout, Federica Fontana, Fennika Huijben, Suze-Anne Korteland, M. Eline Kooi, Paul J. Nederkoorn, Pim A. de Jong, Frank J. Gijsen, Selene Pirola, M. Kamran Ikram, Daniel Bos, Ali C. Akyildiz

**Affiliations:** 1 Department of Epidemiology, Erasmus MC–University Medical Center Rotterdam, 3015 GD Rotterdam, The Netherlands; b.p.berghout@erasmusmc.nl (B.P.B.); d.bos@erasmusmc.nl (D.B.); 2 Department of Radiology and Nuclear Medicine, Erasmus MC–University Medical Center Rotterdam, 3015 GD Rotterdam, The Netherlands; 3 Department of Biomechanical Engineering, Delft University of Technology, 2628 CD Delft, The Netherlands; f.fontana@tudelft.nl (F.F.); fennikahuijben@hotmail.com (F.H.); f.gijsen@erasmusmc.nl (F.J.G.); s.pirola@tudelft.nl (S.P.); 4 Department of Cardiology, Biomedical Engineering, Cardiovascular Institute, Thorax Center, Erasmus MC–University Medical Center Rotterdam, 3015 GD Rotterdam, The Netherlands; s.korteland@erasmusmc.nl; 5 Department of Radiology and Nuclear Medicine, Cardiovascular Research Institute in Maastricht (CARIM), Maastricht University Medical Center+, 6229 ET Maastricht, The Netherlands; eline.kooi@mumc.nl; 6 Department of Neurology, University Medical Center Amsterdam, 1081 HV Amsterdam, The Netherlands; p.j.nederkoorn@amc.uva.nl; 7 Department of Radiology, University Medical Center Utrecht, Utrecht University, 3584 CX Utrecht, The Netherlands; pimdejong@gmail.com; 8 Department of Neurology, Erasmus MC–University Medical Center Rotterdam, 3015 GD Rotterdam, The Netherlands; m.ikram@erasmusmc.nl

**Keywords:** morphometry, vascular calcification, stroke, cerebrovascular disease, cerebral ischemia

## Abstract

**Background**: Intracranial artery calcification detected on CT imaging is a recognized risk factor for ischemic cerebrovascular diseases, but the underlying etiology of this association remains unclear. Differences in objective morphometric characteristics of these calcifications may partially explain this association, yet these measurements are largely absent in the literature. We investigated intracranial artery calcification morphometry in patients with recent anterior ischemic stroke or TIA, assessing potential differences between calcifications in both intracranial carotid arteries (ICAs) located ipsilateral and contralateral to the cerebral ischemia. **Methods**: Among 100 patients (mean age 69.6 (SD 8.8) years) presenting to academic neurology departments, 3D reconstructions of both ICAs were based on clinical CT-angiography images. On these reconstructions, a luminal centerline and cross-sections perpendicular to this centerline were created, facilitating the assessment of calcification morphometry, spatial orientation and stenosis severity. Differences in calcification characteristics between ICAs were assessed using two-sided Wilcoxon signed-rank and χ^2^ tests. **Results**: Among 200 arteries, a median of four (IQR 2–6) individual calcifications were counted, with a mean area of 1.8 (IQR 1.2–2.7) mm^2^, a mean arc width of 43.5 (IQR 32.3–53.2) degrees, and median longitudinal extent of 15.4 (IQR 5.9–27.0) mm. Calcifications were most often present in the anatomical C4 section (56.0%), with predominantly posterosuperior orientation (38.5%) and 42.0% had a local stenosis severity > 70%. None of these aspects significantly differed between ICAs, and this remained after restricting analyses to patients with undetermined etiology. **Conclusions**: We found no differences in morphometrical or spatial aspects of calcifications between ICAs ipsilateral and contralateral to the cerebral ischemia.

## 1. Introduction

Intracranial arteriosclerosis is a recognized risk factor for cerebrovascular diseases such as dementia [1] and stroke [2,3,4]. Research on intracranial arteriosclerosis predominantly focuses on the detection and quantification of its hallmark, intracranial artery calcifications, on CT imaging. Such calcifications are implicated in the development of up to 75% of all strokes [2], raising suggestions that these calcifications may reflect a cause of stroke by themselves [2,5,6], although the etiological mechanism underlying this association remains unclear.

Research on coronary artery calcification suggests that specific morphometric characteristics of arterial calcifications, such as a spherical shape and pericardial-sided location, reflect stable atherosclerotic disease. These stable calcifications are associated with a lower risk of myocardial infarction in the affected arteries when compared to calcifications with less stable morphometric features [7,8]. However, objectively measured morphometric characteristics of intracranial artery calcifications are largely undescribed, precluding further investigations into their exact role in the pathophysiology of cerebrovascular diseases.

Similar to the role of coronary artery calcification in coronary artery disease, investigating morphometric characteristics of intracranial artery calcifications may aid in explaining how it may lead to stroke. Yet, no method currently exists in the literature to objectively measure morphometric characteristics of intracranial artery calcifications. We thus developed a method to perform a detailed morphometric analysis of these calcifications, using data from two cross-sectional studies of patients with recent ischemic stroke or TIA originating from the anterior cerebral circulation. We then assessed differences in morphometric characteristics of ICA calcifications located ipsilaterally and contralaterally to sites of cerebral ischemia, to investigate their role in stroke pathophysiology.

## 2. Materials and Methods

### 2.1. Study Setting

To demonstrate the broad applicability of this method intended for detailed morphometric analyses of intracranial artery calcifications, we applied the method on a random sample of 100 patients with recorded presence of calcifications in both ICAs. To this end, patient data were selected from the prospective multicenter ‘Plaque At RISK’ study (PARISK) cohort study [9], and the cross-sectional registry Erasmus Stroke Study (ESS) [6].

Between 2010 and 2014, the PARISK cohort selected 244 patients from multiple academic medical centers across the Netherlands with recent (diagnosis within 3 months of symptom onset) transient ischemic attack (TIA) or minor stroke. Patients were included based on having ischemia in the territory of a carotid artery with non-severe (<70%) stenosis at baseline, without evidence for a cardiac source of embolism, clotting disorder, severe comorbidity or contraindications for contrast enhanced imaging [9]. Of these, 199 patients underwent baseline multi-detector computed tomography angiography (MDCTA) imaging with a specific focus on carotid bifurcation. An in-depth description of the study design has previously been described in the study-design article [9].

The ESS is a clinical registry study of patients with neurovascular disease admitted to the academic Erasmus Medical Center in Rotterdam, the Netherlands, between 2005 and 2010 [6]. Patients included in the ESS underwent clinical evaluation at presentation in either the outpatient clinic, emergency care department, or neurology ward. In light of routine clinical care, these patients underwent blood sampling and MDCTA imaging. Participants from the ESS were eligible for selection in this study based on the same eligibility criteria used in PARISK [9].

### 2.2. Image Acquisition

In both studies, imaging acquisition was performed using either a 16-, 64- or 128-slice multi-detector row CT system through a standardized optimized contrast-enhanced protocol. The settings for PARISK imaging systems were as follows: 120 kVp, 150–180 reference mAs, collimation 16 × 0.75 mm or 64 × 2 × 0.60 mm, pitch < 1. One center reconstructed 120 kVp images from the 100 kVp and 140 kVp images obtained with dual-energy MDCTA. For ESS scans, the same settings were used, except for a reference mAs of 180–200. All scans ranged from the ascending aorta to the intracranial circulation (3 cm above the sella turcica), with patients receiving 80–85 mL of an iodinated contrast agent (300–320 mg/mL) followed by a 45 mL saline bolus chaser, both at an injection rate of 4 or 5 mL/s. Real-time bolus tracking at the level of the ascending aorta was used for synchronization of the passage of contrast agent and data acquisition.

In PARISK, image reconstructions were made with a field of view of 120–160 mm, matrix size 512 × 512, a slice thickness of 1.00 mm and an increment of 0.60–0.70 mm [9]. In ESS, these same settings were used, except for a slice thickness of 0.75–1.00 mm, and an increment of 0.40–0.60 mm [6].

### 2.3. Image Segmentation Procedure

Segmentations of the ICAs were semi-automatically constructed using the following method. First, MDCTA scans were used as a base input for creating a preliminary 3D geometrical model of the intracranial arteries in the software ‘ITK SNAP’, (version 4.0.2, https://www.itksnap.org/). In ITK SNAP, seeds (bubble contours) were placed in the center of the contrast-enhanced lumens every 3–4 slices, from the opening of the petrous canal to the sella turcica. These seeds were then automatically expanded for 15–25 iterations to fill the entire lumen. To guide this process, a Hounsfield Unit (HU) range of 100–600 was applied to distinguish voxels with intra-arterial contrast from other structures, based on recent literature at the onset of the ESS study [10].

**Figure 1:**
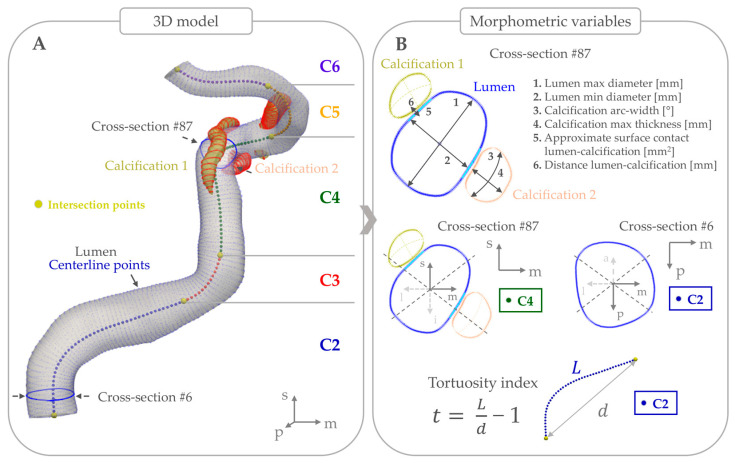
Overview of morphometrical and spatial orientation measurements in a randomly selected left intracranial carotid artery. (**A**) illustrates how the artery is divided in cross-sections, and the identified C2 to C6 sections. (**B**) details the cross-sectional morphometry measurements, the spatial orientation in two different cross-sections, and how the tortuosity index is defined.

This 3D model was then imported into the software ‘3D Slicer’ (version 5.6.1, https://www.slicer.org/) for manual correction of segmentation errors, removal of bony structures, and finalization of the lumen model by smoothing with a factor of 0.3 (Figure 1A). In addition, calcified voxels in proximity to the lumen were identified by manual delineation, using a threshold above 600 HU to distinguish arterial calcification from luminal contrast. The calcified voxels were not smoothened, as this would have caused a loss of morphometric features. This segmentation procedure was carried out on all included scans by trained readers (FF and FH) blinded to anonymized clinical data of patients.

### 2.4. Assessment of Luminal and Calcification Measurements

To obtain morphometric measurements, the final 3D model including both the smoothed lumen and non-smoothed calcifications was exported to an in-house developed MATLAB tool (version 2021b, The MathWorks Inc, Natick, MA, USA). This tool automatically generated luminal centerlines and detailed 2D cross-sectional slices, perpendicular to the centerline, with an inter-slice spacing of 0.2 mm (Figure 1A). These slices highlighted the boundary of the arterial lumen and calcifications on each cross-section, and form the basis of measurements used for analysis.

Furthermore, based on the local curvature of the 3D reconstructed lumen, the C2 through C6 sections of the original Bouthillier classification were automatically identified [11] using a previously validated method [12]. The C2 and C3 segments were considered part of the petrous section, while the C4 through C6 segments were considered non-petrous.

Additionally, radial lines at every 1° originating from the lumen center were generated per slice, enabling the measurement of arterial and calcification metrics per cross-section, as illustrated in the example in Figure 1B. Specifically, the following arterial metrics were measured: (1) lumen diameter and length; (2) tortuosity index ((total artery length/Euclidean distance of the artery endpoints) − 1); (3) tortuosity index of non-petrous segments only, disregarding the C2 and C3 segments; (4) count of individual calcifications; (5) total longitudinal extent of all calcifications; and (6) lumen surface in contact with calcification.

Regarding the morphometrics of calcifications, we assessed the following metrics per slice: (1) calcification area; (2) arc width of calcification; (3) largest distance between luminal and adventitial boundaries of the calcifications, termed calcification thickness; and (4) distance between calcification boundary to lumen boundary.

Besides these morphometrics, the spatial characteristics of these calcifications were measured by automated assessment. This method determined the predominant spatial orientation of the calcification burden along the lateral-medial and anteroinferior-posterosuperior sides of the vessel walls, based on which side contained the highest percentage of total calcification burden. In a similar manner, the predominant location of calcification along the anatomical C2–C6 sections of the artery was identified.

**Figure 2:**
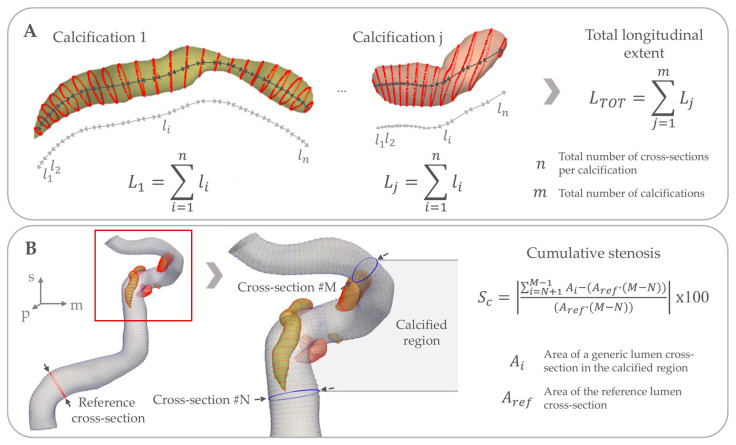
Overview of the morphometrical measurements across multiple cross-sections. (**A**) illustrates the computation of the total longitudinal extent of calcifications. Firstly, the total extent of each calcification $L_{j}$ is computed ($L_{j}$: length of the generic j-th calcification) as the sum of the distance between consecutive calcification contours $l_{i}$ ($l_{i}$: distance between two generic i-th and i+1-th calcification contours). The sum goes from 1 to the total number of cross-sections n of the i-th calcification. Then, the total longitudinal extent $L_{TOT}$ is computed as the sum of the total extent of each calcification $L_{j}$. The sum goes from 1 to the total number of calcifications m. (**B**) illustrates the computation of the cumulative stenosis. Given a reference lumen cross-section with area $A_{ref}$, which is the cross-section with the largest luminal area proximal to the first calcification; the cumulative stenosis $S_{c}$ is computed as the percentage difference between the sum of the lumen cross-sectional areas $A_{i}$ in the calcified region ($A_{i}$: area of the generic i-th lumen cross-section), found between the N-th and M-th cross-sections, and the sum of the lumen areas we would have expected from the largest cross-section prior to the first calcification. This is computed as the product between $A_{ref}$ and the number of cross-sections in the identified calcified region, which is equal to M−N. In this figure, the cumulative stenosis computation for the whole calcified region is presented, but the same strategy can be applied to each calcium body or to a single cross-section.

Finally, as luminal stenosis is clinically the most informative measure in intracranial atherosclerotic disease, we also assessed luminal stenosis due to calcification using the following strategy. We first used the cross-section with the largest luminal area proximal to the first calcification as the reference for comparison. Subsequently, cumulative stenosis due to calcification was first computed as the percentage difference between the sum of the lumen cross-sectional areas in the calcified region, and the sum of the lumen areas we would have expected from the largest cross-section prior to the first calcified region. Following this, we categorized stenosis severity by whether any cross-section with calcification had a stenosis percentage larger than 30%, 50%, 70% or 90%, respectively, when compared to the reference lumen. Figure 2 illustrates the cumulative luminal stenosis due to calcification and total longitudinal extent of calcifications variables, which are the variables measured over multiple cross-sections rather than derived from combining individual cross-sections together.

The analysis was performed on a workstation with an Intel^®^ Xeon^®^ W-2255 processor unit, and 64 GB memory RAM. Computational time for processing each artery was on average 15 min, varying slightly depending on the length of the artery and the total calcification burden.

### 2.5. Patient Characteristics

Demographic and cardiovascular characteristics of patients were retrieved from medical records on admission, with data on ethnic background and smoking behavior being collected by self-report. Hypertension was defined by having a systolic blood pressure ≥ 140 mmHg, a diastolic blood pressure ≥ 90 mmHg, or using any antihypertensive medication. Diabetes was defined by having either a fasting serum glucose > 6.9 mmol/L, a 2 h post-load glucose of >11.0 mmol/L, or the use of any antidiabetic medication. Dyslipidemia was defined by having either a serum total cholesterol ≥ 5.0 mmol/L, or the use of any lipid-lowering medication.

### 2.6. Statistical Analyses

Patient characteristics are presented stratified by the original study and reported through frequencies with percentages or means with standard deviations. All luminal and calcification measurements are presented first for all arteries, and were subsequently stratified by ipsilateral and contralateral sides based on the site of ischemia, through medians with interquartile ranges (IQR) or frequencies with percentages. Possible two-sided differences in any of these measurements between arteries were assessed by paired Wilcoxon signed-rank tests for count or continuous variables with non-normal distributions, or χ^2^-test for categorical variables, with α set to 0.05. As a sensitivity analysis, we assessed differences in measurements between arteries in patients with no other known etiology for their ischemic event by restricting the study to patients with undetermined etiology and repeating the analyses.

To assess inter-rater agreement of this new method for extracting lumen and calcification morphometrics, three researchers (BPB, 6 years of experience in assessing intracranial arterial structures; FF, 2 years of experience; and FH, 1 year of experience) repeated segmentations of 10 ICAs from five patients, after which the absolute agreement between these raters was established by calculating two-way mixed effects intraclass correlation coefficients (ICCs) and 95% confidence intervals (CI) per measurement.

All statistical tests were performed in R (version 4.2.2, R Foundation for Statistical Computing, Vienna, Austria) with the ‘irr’ (v0.84.1) package.

## 3. Results

### 3.1. Study Characteristics

An overview of the demographic and clinical characteristics of the 100 included patients, stratified by study type, is provided in Table 1. The mean age of participants at inclusion was 69.6 (SD 8.8) years, and 35.0% were female. The right side was symptomatic in 53 patients and 40 patients suffered a TIA.

### 3.2. Morphometry and Comparisons Between Sides Ipsi- and Contralateral to the Cerebral Ischemia

Summaries of the measurements in 200 arteries are provided in Table 2. The minimum and maximum lumen diameter ranged between 2.2 (IQR 1.9–2.5) and 5.9 (IQR 5.4–6.4) mm, with a median tortuosity index of 0.7 (IQR 0.6–0.8). A median of 4 (IQR 2–6) individual calcifications were counted among all arteries, with a mean area of 1.8 (IQR 1.2–2.7) mm^2^, a mean arc width of 43.5 (IQR 32.3–53.2) degrees, a mean thickness of 1.2 (IQR 1.0–1.4) mm, and median total longitudinal extent of 16.2 (IQR 7.6–26.3) mm. The mean distance between the boundary of calcifications to the luminal boundary was 0.3 (IQR 0.2–0.4) mm and the calcifications had a mean surface contact area with the lumen of 12.8 (IQR 3.0–26.3) mm^2^.

The bulk of calcifications were predominantly situated in the C4 segments (56.0%), most often with a posterosuperior orientation to the lumen center (38.5%). The maximum cumulative stenosis detected due to these calcifications had a median of 55.5% (39.1–66.6%), where 42% of calcifications had cross-sections with over 70% stenosis when compared with the lumen reference diameter.

**Figure 3:**
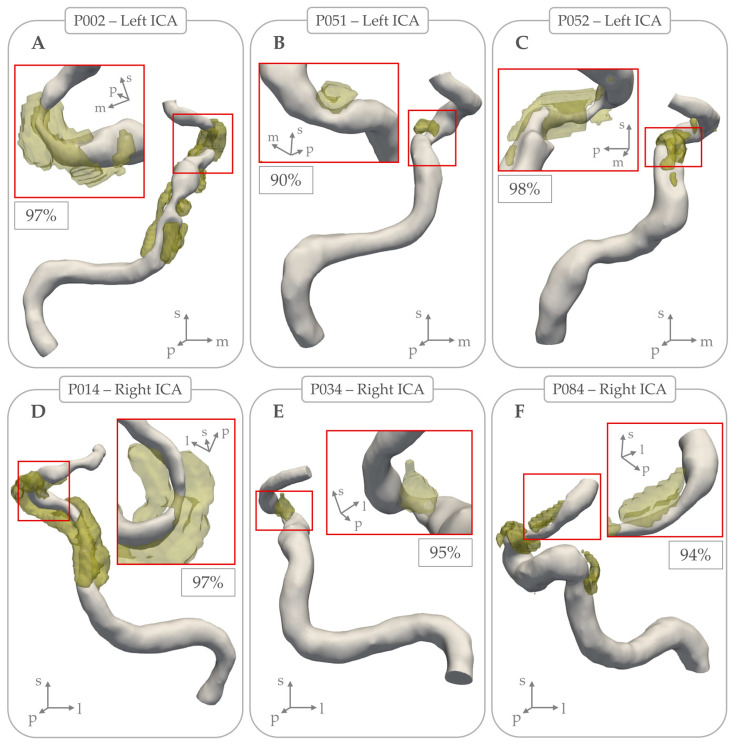
Examples of intracranial carotid arteries where arterial calcifications (in yellow) coincided with local lumen stenosis percentage > 90%. Subpanels (**A**), (**B**), and (**C**) show examples of 3 left intracranial carotid arteries, with local stenosis lumen percentage of 97%, 90%, and 98% respectively. Subpanels (**D**), (**E**), and (**F**) show examples of 3 right intracranial carotid arteries, with local stenosis lumen percentage of 97%, 95%, and 94% respectively.

Figure 3 shows three examples where calcifications caused >90% lumen stenosis. Table 2 provides an overview of measurements with stratifications and statistical testing for differences per ICA located ipsilateral and contralateral to the side of the ischemia. This comparison revealed no significant statistical differences in any morphometric characteristics related to lumen or calcification. These results did not change after restricting analyses to patients with undetermined etiology for their stroke.

### 3.3. Inter-Rater Agreement

Table 3 provides an overview of ICCs depicting the absolute agreement in morphometric characteristics from 10 ICAs measured by three raters. In general, there was high agreement in artery and calcification metrics with ICCs ranging between 0.57–1.00, except for the maximum lumen diameter (ICC 0.27, 95% CI [−0.02|0.65]) and the distance between calcification and lumen boundaries (ICC 0.25, 95% CI [−0.10|0.68]).

## 4. Discussion

In this cross-sectional study of patients with recent ischemic stroke of the anterior cerebral circulation, we described objective morphometric characteristics of IAC based on clinical CTA images. Notably, our analyses revealed no significant differences in any of these characteristics between ICAs ipsilateral and contralateral to the side of cerebral ischemia. Furthermore, we found the majority of ICA calcification was located in the C4 segment, and ICA calcifications predominantly had a posterosuperior orientation.

Comparing our morphometrical measurements of intracranial artery calcification with existing literature is challenging, as most descriptions of these calcifications are limited to either morphological assessments -rather than morphometrical- or general estimations of presence and volume. Two morphological subtypes of ICA calcification may be distinguished using a subjective non-contrast CT scoring rule [13,14]. These subtypes have been extensively investigated for their effects on cerebrovascular disease pathophysiology [15]. The internal elastic lamina type, characterized by continuous, circumferential, and thin calcification around the ICA, is associated with an increased risk of stroke in general [3] and low intracranial collateral status [16]. In contrast, the intimal type calcification, characterized by singular thick calcifications reflecting classical atherosclerotic disease [15], has been associated with lacunar stroke [17] and an increased collateral formation [16]. The non-contrast CT based scoring rule used to distinguish these subtypes shows adequate inter-rater agreement (Kappa 0.73–0.89) [15]. Our method allows for objective, semi-automated and accurate assessment of intracranial artery calcification morphometry, thereby providing an advantage over the existing subjective methods to describe these calcifications.

This study did not detect significant differences in individual morphometrical or spatial aspects of calcifications between ICAs ipsilateral and contralateral to the cerebral ischemia. This finding is noteworthy given the extensive literature suggesting their involvement in cerebrovascular disease pathophysiology [2,6,16,17,18,19]. A lack of investigations regarding vascular territory-specific effects from intracranial artery calcifications has been suggested as a significant gap in the literature [20]. We specifically aimed to fill this gap by examining these effects in this study of patients with recent anterior cerebral ischemia of predominantly undetermined etiology. While our findings do not support the hypothesis that ICA calcifications are causally involved with cerebrovascular pathophysiology, the cross-sectional design of this study limits our ability to properly determine these effects. Alternatively, it may be that individual morphometrical aspects alone hold no value in explaining previous associations reported in the literature. Rather, combinations of individual aspects may be applied to detect potential novel calcification types that may reflect different stages of arteriosclerotic disease.

Our findings underscore the need for future longitudinal studies to explore the role of intracranial artery calcifications in cerebrovascular disease pathophysiology. Such studies should ideally combine contrast-based and non-contrast CT data to determine the diagnostic accuracy and added benefit of combined morphometrical and spatial characteristics in distinguishing morphological subtypes. This approach would be especially valuable in ambiguous cases where the existing scoring rule [15] does not clearly differentiate between these subtypes. Alternatively, future studies may find merit in investigating calcification morphometry in other vessel beds. Specifically, certain morphological features of coronary artery calcification have been suggested to be associated with a reduced risk of cardiovascular disease [7]. Given that calcification in coronary arteries is known to correlate with intracranial artery calcification [21,22,23], investigating the morphometrical and spatial characteristics of coronary artery calcification could reveal more insights into its relationship with ischemic heart disease.

Furthermore, we report novel findings regarding the predominant location and spatial orientation of ICA calcifications. The majority of the calcification burden predominantly resided in the C4 sections. Perhaps this is due to its length, as it was the longest of the five sections evaluated, although notably the shorter C5 section also exhibited a significant calcification burden. Alternatively, these two sections display a considerable tortuosity, which could have contributed to this particular distribution. Conversely, the C2 and C3 sections, which are encased by the carotid canal, rarely exhibited major calcification. This is possibly due to dampening of the surrounding bone against the impaired arterial distension effects that drive arteriosclerotic processes [24]. To our knowledge, only Fisher and colleagues have previously described the spatial orientation of ICA calcifications in their study published in 1965, where they mention that calcification depositions were equally present in both inner and outer curves among the ICA siphons [25]. However, any direct comparison between this histopathological study and our imaging-based study is complicated by differences in calcification assessment and underlying study design.

### Strengths and Limitations

A key strength of this study is the selection of patients with ischemic stroke of predominantly undetermined etiology, who underwent an extensive diagnostic workup in academic medical institutions. This ensures that the presence of intracranial artery calcification is more likely related to the underlying cause of the cerebral ischemia. Another strength is that these specific morphometrical characteristics are based on established markers from coronary calcification studies [7,26,27]. This method thus provides an objective method to measure arterial calcification using established markers of shape, beyond existing subjective visual scoring methods used on non-contrast imaging [15,28]. Finally, all calcification metrics demonstrated high interrater agreement, indicating reliability across varying levels of radiological expertise. Although admittedly the measured luminal aspects showed poor interrater agreement, they did align well with established values in the literature (mean internal carotid artery diameter = 4.53 mm, SD 0.47 [29]).

Despite these strengths, several important limitations need to be mentioned. The primary limitation is the requirement of a high threshold of 600 HU in order to differentiate arterial calcifications from luminal contrast, restricting the detection to high-density calcifications only [10]. This may have led to the misclassification of arteries as having no IAC when low-density calcifications were present, potentially limiting our sample to more dense calcifications which are associated with more stable arteriosclerotic disease. Furthermore, thin internal elastic lamina calcifications may be a cause of unexplained stroke and these can remain undetected on CTA. However, contrast-enhanced images allow for more accurate differentiation of arterial calcifications from other bony intracranial structures like dural calcifications or bony protrusions from the carotid canal, ensuring that the measured calcifications were indeed arterial calcifications. Another limitation is the cross-sectional design, which precludes the assessment of longitudinal changes to these calcifications and association analyses of recurrent stroke risk. Although PARISK does have longitudinal data available, we could not study these aspects in patients selected from the ESS. Finally, the generalizability of this study is limited by the predominant selection of patients of Western European descent. The prevalence of IAC differs widely among different ethnic populations [4], and our measurements may thus not be entirely representative of other ethnicities. The evaluation of the generalizability of this study is limited further by the lack of structured data on in-depth symptomatology, treatment provided for the ischemic stroke events and functional outcomes. Furthermore, these results only reflect patients with calcifications in both ICAs.

## 5. Conclusions

We developed a semi-automatic method for objectively measuring the morphometry of intracranial artery calcifications on CTA imaging. These measurements can be used in biomechanical modeling studies and for the objective discrimination of specific intracranial artery calcification subtypes, potentially improving risk stratification in studies on intracranial arteriosclerotic diseases. Our initial application suggests that individual morphometric aspects of these calcifications are unrelated to the side of symptoms. However, further longitudinal research is needed to confirm this finding.

## Figures and Tables

**Table 1:** Characteristics of the study population (N = 100), stratified by cohort.

Characteristic	N/Mean (%/SD)		*p*-Value ^1^
	*Study*		
	ESS	PARISK	
N	50 (50.0)	50 (50.0)	-
Age, years	71.0 (10.2)	68.3 (7.1)	0.13
Sex, female	24 (48.0)	11 (22.0)	0.01
Ethnicity, Caucasian	45 (90.0)	48 (96.0)	0.43
Smoking behavior			
Never	9 (19.1)	8 (16.0)	0.30
Former	21 (44.7)	30 (60.0)	
Current	17 (36.2)	12 (24.0)	
Hypertension	36 (72.0)	35 (83.3)	0.30
Diabetes Mellitus, type II	20 (40.0)	8 (17.8)	0.03
Dyslipidemia	45 (90.0)	41 (83.7)	0.53
History of cardiovascular disease	32 (64.0)	28 (57.1)	0.62
History of ischemic cardiovascular disease	25 (50.0)	26 (52.0)	1.00
Use of any anti-thrombotic medication	38 (76.0)	25 (50.0)	0.01
TOAST			<0.001
Large artery atherosclerosis	20 (40.0)	2 (4.0)	
Small vessel occlusion	8 (16.0)	0 (0.0)	
Undetermined etiology	22 (44.0)	48 (96.0)	

Abbreviations: ESS = Erasmus Stroke Study, PARISK = Plaque At RISK, N = count, SD = standard deviation, TOAST = Trial of Org 10172 in Acute Stroke Treatment. ^1^ *p*-values were established by comparative tests between study cohorts, using Students’ *t*-test for continuous data, or Chi-squared test for categorical data.

**Table 2:** Morphometrical measurements of arterial lumens and calcifications.

Measurement	Total	Ipsilateral	Contralateral
*Count, N*	*200*	*100*	*100*
Maximum lumen diameter, mm	5.9 [5.4, 6.4]	5.8 [5.4, 6.4]	5.9 [5.3, 6.4]
Mean lumen diameter, mm	4.0 [3.7, 4.4]	4.0 [3.7, 4.4]	4.0 [3.8, 4.4]
Minimum lumen diameter, mm	2.2 [1.9, 2.5]	2.3 [1.9, 2.5]	2.2 [1.8, 2.5]
Total measured artery length, mm	75.4 [70.7, 80.8]	75.4 [71.5, 79.6]	75.5 [69.8, 81.1]
Arterial tortuosity index	0.7 [0.6, 0.8]	0.7 [0.6, 0.8]	0.7 [0.6, 0.9]
Non-petrous arterial tortuosity index	0.8 [0.7, 1.0]	0.8 [0.6, 0.9]	0.8 [0.7, 1.0]
Total longitudinal extent of calcifications, mm	16.2 [7.6, 26.3]	16.2 [8.1, 25.4]	16.1 [6.8, 27.0]
Calcification count, n	4.0 [2.0, 6.0]	4.0 [2.0, 6.0]	4.0 [2.0, 6.0]
Total surface contact of lumen with calcification, mm^2^	12.8 [3.0, 26.3]	11.9 [3.1, 26.5]	13.8 [2.8, 26.0]
Mean distance from calcification to lumen boundary, mm	0.3 [0.2, 0.4]	0.3 [0.2, 0.4]	0.3 [0.2, 0.4]
Maximum calcification area, mm^2^	3.9 [2.7, 6.6]	3.9 [2.7, 7.2]	3.9 [2.7, 6.3]
Mean calcification area, mm^2^	1.8 [1.2, 2.7]	1.7 [1.2, 2.8]	1.9 [1.3, 2.7]
Maximum calcification arc width, °	80.0 [58.0, 117.3]	81.0 [53.0, 121.5]	77.0 [62.5, 115.5]
Mean calcification arc width, °	43.5 [32.3, 53.2]	41.2 [32.1, 52.9]	45.4 [33.9, 53.4]
Maximum calcification thickness, mm	1.9 [1.5, 2.4]	1.9 [1.6, 2.3]	1.9 [1.5, 2.4]
Mean calcification thickness, mm	1.2 [1.0, 1.4]	1.2 [1.0, 1.4]	1.2 [0.9, 1.4]
Maximum calcification area, mm^2^	3.9 [2.7, 6.6]	3.9 [2.7, 7.2]	3.9 [2.7, 6.3]
Maximum cumulative stenosis from calcification, %	55.5 [39.1, 66.6]	55.4 [39.3, 67.9]	56.5 [39.0, 65.3]
Highest stenosis severity due to calcification, N			
30–50%	42 (21.0)	20 (20.0)	22 (22.0)
50–70%	61 (30.5)	32 (32.0)	29 (29.0)
70–90%	75 (37.5)	39 (39.0)	36 (36.0)
>90%	9 (4.5)	3 (3.0)	6 (6.0)
Predominant location of calcifications, per anatomical section			
C2	5 (2.5)	4 (4.0)	1 (1.0)
C3	5 (2.5)	2 (2.0)	3 (3.0)
C4	112 (56.0)	56 (56.0)	56 (56.0)
C5	47 (23.5)	22 (22.0)	25 (25.0)
C6	31 (15.5)	16 (16.0)	15 (15.0)
Predominant orientation of calcifications to the sagittal plane			
Medial	17 (8.5)	6 (6.0)	11 (11.0)
Lateral	49 (24.5)	26 (26.0)	23 (23.0)
Anteroinferior	57 (28.5)	32 (32.0)	25 (25.0)
Posterosuperior	77 (38.5)	36 (36.0)	41 (41.0)

Continuous values are presented for the whole sample in medians and the interquartile ranges in brackets. *p*-values were derived from two-sided Wilcoxon sign rank tests or χ^2^-tests conducted between the arteries located ipsilaterally and contralaterally to the infarction, using an α of 0.05.

**Table 3:** Intraclass correlation coefficients depicting the inter-rater absolute agreement for morphometrical measurements derived through this method.

Measurement	ICC ^1^	95% Confidence Interval
Maximum lumen diameter	0.27	−0.02|0.65
Mean lumen diameter	0.59	0.15|0.86
Minimum lumen diameter	0.57	0.21|0.85
Total measured artery length	0.78	0.29|0.94
Arterial tortuosity index	0.87	0.60|0.96
Non-petrous arterial tortuosity index	0.90	0.74|0.97
Total longitudinal extent of calcifications	0.99	0.99|1.00
Calcification count	0.90	0.74|0.97
Total surface contact of lumen with calcification	0.71	0.37|0.91
Mean distance from calcification to lumen boundary	0.25	−0.10|0.68
Maximum calcification area	0.99	0.97|1.00
Mean calcification area	1.00	0.99|1.00
Maximum calcification arcwidth	0.98	0.94|0.99
Mean calcification arcwidth	0.99	0.96|1.00
Maximum calcification thickness	0.90	0.74|0.97
Mean calcification thickness	0.97	0.92|0.99

Coefficients were estimated based on measurements on ten ICAs from five randomly selected patients, based on three raters (BPB, FF & FH). ^1^ Intraclass correlation coefficient.

## Data Availability

The anonymized data presented in this study are available upon reasonable request to the corresponding author.

## Abbreviations

The following abbreviations are used in this manuscript:

CT	Computed Tomography
TIA	Transient Ischemic Attack
IAC	Intracranial Artery Calcification
ICA	Intracranial Carotid Artery
SD	Standard Deviation
IQR	Inter Quartile Range
PARISK	Plaque At RISK
ESS	Erasmus Stroke Study
MDCTA	Multi-Detector Computed Tomography Angiography
HU	Hounsfield Unit
ICC	Intraclass Correlation Coefficient
CI	Confidence Interval
